# Diacetato-κ*O*;κ^2^ *O*,*O*′-aqua­(2,4,6-tri-2-pyridyl-1,3,5-triazine-κ^3^ *N* ^2^,*N* ^1^,*N* ^6^)manganese(II) monohydrate

**DOI:** 10.1107/S1600536811032016

**Published:** 2011-08-17

**Authors:** Kwang Ha

**Affiliations:** a School of Applied Chemical Engineering, The Research Institute of Catalysis, Chonnam National University, Gwangju 500-757, Republic of Korea

## Abstract

The Mn^II^ ion in the title compound, [Mn(CH_3_CO_2_)_2_(C_18_H_12_N_6_)(H_2_O)]·H_2_O, is seven-coordinated in an approximately penta­gonal–bipyramidal geometry by three N atoms of the tridentate 2,4,6-tri-2-pyridyl-1,3,5-triazine ligand and four O atoms from two distinct anionic acetato ligands and a water mol­ecule. One acetate anion chelates the Mn atom *via* two O atoms occupying equatorial positions, and the other anion coordinates the Mn atom as a monodentate ligand *via* one O atom. The complex and solvent water mol­ecules are linked by inter- and intra­molecular O—H⋯O, O—H⋯N and C—H⋯O hydrogen bonds into a three-dimensional network.

## Related literature

For the crystal structure of 2,4,6-tri-2-pyridyl-1,3,5-triazine (tptz), see: Drew *et al.* (1998 ▶). For tptz complexes with a five-coordinate Mn(II) atom, see: Ha (2010 ▶), and with a seven-coordinate Mn(II) atom, see: Majumder *et al.* (2006 ▶); Zhang *et al.* (2008 ▶); Lo & Ng (2009 ▶).
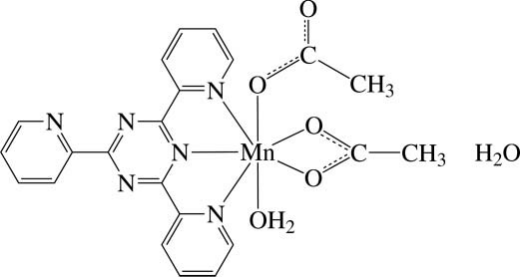


## Experimental

### 

#### Crystal data

[Mn(C_2_H_3_O_2_)_2_(C_18_H_12_N_6_)(H_2_O)]·H_2_O
*M* *_r_* = 521.40
Monoclinic, 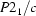

*a* = 10.341 (2) Å
*b* = 24.977 (5) Å
*c* = 9.8284 (19) Å
β = 118.073 (4)°
*V* = 2239.9 (8) Å^3^
*Z* = 4
Mo *K*α radiation
μ = 0.64 mm^−1^
*T* = 200 K
0.32 × 0.24 × 0.17 mm

#### Data collection

Bruker SMART 1000 CCD diffractometer
Absorption correction: multi-scan (*SADABS*; Bruker, 2000 ▶) *T*_min_ = 0.863, *T*_max_ = 1.000
16577 measured reflections
5548 independent reflections
3076 reflections with *I* > 2σ(*I*)
*R*_int_ = 0.068

#### Refinement

*R*[*F* ^2^ > 2σ(*F* ^2^)] = 0.056
*wR*(*F* ^2^) = 0.153
*S* = 1.00
5548 reflections
318 parameters
H-atom parameters constrained
Δρ_max_ = 0.49 e Å^−3^
Δρ_min_ = −0.49 e Å^−3^

### 

Data collection: *SMART* (Bruker, 2000 ▶); cell refinement: *SAINT* (Bruker, 2000 ▶); data reduction: *SAINT*; program(s) used to solve structure: *SHELXS97* (Sheldrick, 2008 ▶); program(s) used to refine structure: *SHELXL97* (Sheldrick, 2008 ▶); molecular graphics: *ORTEP-3* (Farrugia, 1997 ▶) and *PLATON* (Spek, 2009 ▶); software used to prepare material for publication: *SHELXL97*.

## Supplementary Material

Crystal structure: contains datablock(s) global. DOI: 10.1107/S1600536811032016/ng5206sup1.cif

Additional supplementary materials:  crystallographic information; 3D view; checkCIF report

## Figures and Tables

**Table:** 

Mn1—O3	2.113 (2)
Mn1—O5	2.245 (2)
Mn1—O1	2.284 (2)
Mn1—O2	2.295 (2)
Mn1—N1	2.298 (3)
Mn1—N4	2.387 (3)
Mn1—N6	2.393 (3)

**Table:** 

O3—Mn1—O5	169.93 (10)
O1—Mn1—O2	57.06 (8)
N1—Mn1—N4	68.35 (9)
N1—Mn1—N6	68.43 (9)

**Table 2:** Hydrogen-bond geometry (Å, °)

*D*—H⋯*A*	*D*—H	H⋯*A*	*D*⋯*A*	*D*—H⋯*A*
O5—H5*A*⋯N5^i^	0.84	2.16	2.924 (3)	151
O5—H5*B*⋯O6^ii^	0.84	1.88	2.704 (3)	168
O6—H6*A*⋯O2	0.84	1.95	2.791 (3)	175
O6—H6*B*⋯O4^iii^	0.84	1.87	2.711 (4)	176
C3—H3⋯O5^iv^	0.95	2.46	3.399 (4)	170
C5—H5⋯O3^i^	0.95	2.59	3.338 (4)	136
C6—H6⋯O1	0.95	2.33	2.983 (4)	125
C18—H18⋯O2	0.95	2.55	3.198 (4)	125

Symmetry codes: (i) 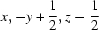; (ii) 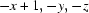; (iii) 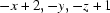; (iv) 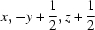.

**Fig. 1.**
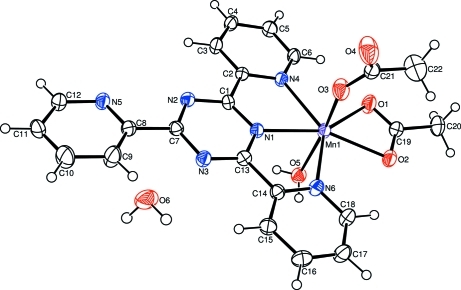
The structure of the title compound, with displacement ellipsoids drawn at the 40% probability level; H atoms are shown as small circles of arbitrary radius.

**Fig. 2.**
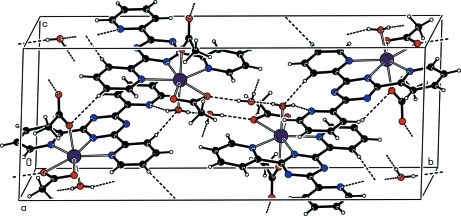
View of the unit-cell contents of the title compound. Hydrogen-bond interactions are drawn with dashed lines.

## supplementary crystallographic information

### Comment

The X-ray crystal structures of 2,4,6-tri-2-pyridyl-1,3,5-triazine (tptz) (Drew *et al.* 1998) and five- or seven-coordinated Mn(II)-tptz complexes, such as [MnCl_2_(tptz)] (Ha, 2010), [Mn(C_2_H_3_O_2_)(C_2_N_3_)(tptz)(H_2_O)].(H_2_O)_2_ (Majumder *et al.*, 2006; Zhang *et al.*, 2008) and [Mn(C_2_F_3_O_2_)(tptz)(H_2_O)_2_]C_2_F_3_O_2_ (Lo & Ng, 2009), have been investigated previously.

The title compound consists of the neutral Mn(II) complex [Mn(C_2_H_3_O_2_)_2_(tptz)(H_2_O)] and a solvent water molecule. In the reaction of Mn(CH_3_CO_2_)_3_.2H_2_O with tptz, it seems that the Mn^III^ ion reduced to the Mn^II^ ion. In the complex, the Mn^II^ ion is seven-coordinated in an approximately pentagonal-bipyramidal geometry by three N atoms of the tridentate tptz ligand and four O atoms from two distinct anionic acetato ligands and a water molecule (Fig. 1). The coordination modes of the acetate anions are quite different: one anion chelates the Mn atom *via* two O atoms occupying equatorial positions, and the other anion coordinates the Mn atom as a monodentate ligand *via* one O atom and occupies the axial sites together with the water ligand. The Mn—O and Mn—N bond lengths are somewhat different, respectively (Table 1). The Mn1—N4/6(pyridyl) bonds are somewhat longer than the Mn1—N1(triazine) bond, and the Mn1—O1/2(equatorial) bonds are slightly longer than the Mn1—O3/5(axial) bonds. The O1—Mn1—O2 chelating angle is considerably smaller than the N1—Mn1—N4/6 chelating angles and the apical O3—Mn1—O5 bond is slightly bent with a bond angle of 169.93 (10)°. The carboxylate groups of the anionic ligands appear to be delocalized on the basis of the C—O bond lengths [C—O: 1.235 (4)–1.269 (4) Å]. In the crystal, the two pyridyl rings coordinated to the Mn atom are located approximately parallel to their carrier triazine ring, making dihedral angles of 1.9 (2)° and 2.8 (2)°. The dihedral angle between the uncoordinated pyridyl ring and triazine ring is 7.8 (2)°. The complex and solvent water molecules are linked by inter- and intramolecular O—H···O, O—H···N and C—H···O hydrogen bonds into a three-dimensional network (Fig. 2 and Table 2). The compounds stack in columns along the [101] direction and display numerous intermolecular π-π interactions between the six-membered rings, with a shortest centroid-centroid distance of 3.493 (2) Å.

### Experimental

To a solution of Mn(CH_3_CO_2_)_3_.2H_2_O (0.4022 g, 1.50 mmol) in MeOH (30 ml) was added 2,4,6-tri-2-pyridyl-1,3,5-triazine (0.1561 g, 0.50 mmol) and stirred for 3 h at room temperature. After removal of the formed dark brown precipitate by filtration, the solvent of the filtrate was evaporated, and the residue was washed with acetone and dried under vacuum, to give a yellow powder (0.3207 g). Crystals suitable for X-ray analysis were obtained by slow evaporation from a CH_3_NO_2_/MeOH solution.

### Refinement

Carbon-bound H atoms were positioned geometrically and allowed to ride on their respective parent atoms [C—H = 0.95 Å (CH) or 0.98 Å (CH_3_) and *U*_iso_(H) = 1.2*U*_eq_(C) or 1.5*U*_eq_(methyl C)]. The H atoms of the water ligand and solvent molecule were located from Fourier difference maps then allowed to ride on their parent O atoms in the final cycles of refinement with O—H = 0.84 Å and *U*_iso_(H) = 1.5 *U*_eq_(O).

### Crystal data

**Table:**

[Mn(C_2_H_3_O_2_)_2_(C_18_H_12_N_6_)(H_2_O)]·H_2_O	*F*(000) = 1076
*M**_r_* = 521.40	*D*_x_ = 1.546 Mg m^−^^3^
Monoclinic, *P*2_1_/*c*	Mo *K*α radiation, λ = 0.71073 Å
Hall symbol: -P 2ybc	Cell parameters from 3469 reflections
*a* = 10.341 (2) Å	θ = 2.2–27.2°
*b* = 24.977 (5) Å	µ = 0.64 mm^−^^1^
*c* = 9.8284 (19) Å	*T* = 200 K
β = 118.073 (4)°	Block, yellow
*V* = 2239.9 (8) Å^3^	0.32 × 0.24 × 0.17 mm
*Z* = 4	

### Data collection

**Table:**

Bruker SMART 1000 CCD diffractometer	5548 independent reflections
Radiation source: fine-focus sealed tube	3076 reflections with *I* > 2σ(*I*)
graphite	*R*_int_ = 0.068
φ and ω scans	θ_max_ = 28.3°, θ_min_ = 2.2°
Absorption correction: multi-scan (*SADABS*; Bruker, 2000)	*h* = −11→13
*T*_min_ = 0.863, *T*_max_ = 1.000	*k* = −28→33
16577 measured reflections	*l* = −13→13

### Refinement

**Table:**

Refinement on *F*^2^	Primary atom site location: structure-invariant direct methods
Least-squares matrix: full	Secondary atom site location: difference Fourier map
*R*[*F*^2^ > 2σ(*F*^2^)] = 0.056	Hydrogen site location: inferred from neighbouring sites
*wR*(*F*^2^) = 0.153	H-atom parameters constrained
*S* = 1.00	*w* = 1/[σ^2^(*F*_o_^2^) + (0.0615*P*)^2^] where *P* = (*F*_o_^2^ + 2*F*_c_^2^)/3
5548 reflections	(Δ/σ)_max_ < 0.001
318 parameters	Δρ_max_ = 0.49 e Å^−^^3^
0 restraints	Δρ_min_ = −0.49 e Å^−^^3^

### Special details

**Table:**

Geometry. All e.s.d.'s (except the e.s.d. in the dihedral angle between two l.s. planes) are estimated using the full covariance matrix. The cell e.s.d.'s are taken into account individually in the estimation of e.s.d.'s in distances, angles and torsion angles; correlations between e.s.d.'s in cell parameters are only used when they are defined by crystal symmetry. An approximate (isotropic) treatment of cell e.s.d.'s is used for estimating e.s.d.'s involving l.s. planes.
Refinement. Refinement of *F*^2^ against ALL reflections. The weighted *R*-factor *wR* and goodness of fit *S* are based on *F*^2^, conventional *R*-factors *R* are based on *F*, with *F* set to zero for negative *F*^2^. The threshold expression of *F*^2^ > σ(*F*^2^) is used only for calculating *R*-factors(gt) *etc*. and is not relevant to the choice of reflections for refinement. *R*-factors based on *F*^2^ are statistically about twice as large as those based on *F*, and *R*- factors based on ALL data will be even larger.

### Fractional atomic coordinates and isotropic or equivalent isotropic displacement parameters (Å^2^)

**Table:**

	*x*	*y*	*z*	*U*_iso_*/*U*_eq_	
Mn1	0.66399 (5)	0.120956 (18)	0.21934 (6)	0.02892 (17)	
O1	0.8310 (3)	0.12897 (9)	0.1302 (3)	0.0428 (6)	
O2	0.7412 (3)	0.04962 (9)	0.1291 (3)	0.0378 (6)	
O3	0.8167 (3)	0.11251 (10)	0.4552 (3)	0.0500 (7)	
O4	0.9755 (3)	0.09035 (13)	0.6912 (3)	0.0693 (9)	
O5	0.4723 (2)	0.13074 (8)	−0.0166 (2)	0.0333 (6)	
H5A	0.4289	0.1603	−0.0328	0.050*	
H5B	0.4083	0.1066	−0.0473	0.050*	
N1	0.5198 (3)	0.16134 (10)	0.3098 (3)	0.0262 (6)	
N2	0.4431 (3)	0.23975 (10)	0.3854 (3)	0.0286 (6)	
N3	0.3510 (3)	0.15448 (10)	0.4067 (3)	0.0297 (6)	
N4	0.6929 (3)	0.21595 (10)	0.2323 (3)	0.0278 (6)	
N5	0.2620 (3)	0.28877 (11)	0.4719 (3)	0.0319 (7)	
N6	0.5166 (3)	0.05706 (10)	0.2655 (3)	0.0302 (6)	
C1	0.5225 (3)	0.21397 (12)	0.3310 (3)	0.0244 (7)	
C2	0.6177 (3)	0.24544 (12)	0.2864 (3)	0.0254 (7)	
C3	0.6272 (4)	0.30056 (12)	0.2985 (4)	0.0313 (8)	
H3	0.5735	0.3199	0.3384	0.038*	
C4	0.7163 (4)	0.32668 (13)	0.2514 (4)	0.0343 (8)	
H4	0.7255	0.3645	0.2590	0.041*	
C5	0.7919 (4)	0.29748 (13)	0.1930 (4)	0.0351 (8)	
H5	0.8521	0.3149	0.1575	0.042*	
C6	0.7786 (4)	0.24225 (13)	0.1872 (4)	0.0313 (8)	
H6	0.8328	0.2222	0.1493	0.038*	
C7	0.3586 (3)	0.20828 (12)	0.4201 (3)	0.0266 (7)	
C8	0.2631 (4)	0.23502 (13)	0.4750 (4)	0.0295 (7)	
C9	0.1796 (4)	0.20573 (15)	0.5232 (5)	0.0486 (10)	
H9	0.1846	0.1677	0.5267	0.058*	
C10	0.0883 (5)	0.23274 (16)	0.5664 (5)	0.0549 (11)	
H10	0.0292	0.2134	0.5997	0.066*	
C11	0.0831 (4)	0.28754 (15)	0.5611 (4)	0.0385 (9)	
H11	0.0202	0.3069	0.5893	0.046*	
C12	0.1723 (4)	0.31367 (14)	0.5133 (4)	0.0354 (8)	
H12	0.1695	0.3517	0.5099	0.042*	
C13	0.4331 (3)	0.13336 (12)	0.3489 (4)	0.0260 (7)	
C14	0.4284 (3)	0.07435 (12)	0.3221 (3)	0.0276 (7)	
C15	0.3382 (4)	0.04070 (13)	0.3515 (4)	0.0326 (8)	
H15	0.2775	0.0544	0.3918	0.039*	
C16	0.3381 (4)	−0.01338 (13)	0.3210 (4)	0.0389 (9)	
H16	0.2770	−0.0375	0.3394	0.047*	
C17	0.4277 (4)	−0.03147 (14)	0.2638 (4)	0.0385 (9)	
H17	0.4304	−0.0684	0.2426	0.046*	
C18	0.5144 (4)	0.00486 (13)	0.2373 (4)	0.0353 (8)	
H18	0.5756	−0.0082	0.1967	0.042*	
C19	0.8296 (4)	0.07960 (14)	0.1086 (4)	0.0331 (8)	
C20	0.9353 (4)	0.05507 (15)	0.0623 (5)	0.0493 (10)	
H20A	1.0283	0.0478	0.1546	0.074*	
H20B	0.9522	0.0799	−0.0050	0.074*	
H20C	0.8945	0.0215	0.0070	0.074*	
C21	0.9334 (4)	0.09139 (13)	0.5510 (4)	0.0360 (9)	
C22	1.0344 (5)	0.06987 (19)	0.4916 (5)	0.0757 (14)	
H22A	1.1331	0.0653	0.5780	0.114*	
H22B	1.0378	0.0951	0.4171	0.114*	
H22C	0.9976	0.0352	0.4413	0.114*	
O6	0.7515 (3)	−0.06096 (9)	0.0946 (3)	0.0496 (7)	
H6A	0.7506	−0.0275	0.1014	0.074*	
H6B	0.8347	−0.0702	0.1638	0.074*	

### Atomic displacement parameters (Å^2^)

**Table:**

	*U*^11^	*U*^22^	*U*^33^	*U*^12^	*U*^13^	*U*^23^
Mn1	0.0317 (3)	0.0250 (3)	0.0342 (3)	0.0026 (2)	0.0189 (2)	−0.0010 (2)
O1	0.0490 (16)	0.0322 (15)	0.0636 (17)	0.0011 (11)	0.0400 (14)	−0.0048 (12)
O2	0.0395 (15)	0.0305 (13)	0.0502 (15)	0.0025 (10)	0.0267 (13)	−0.0055 (11)
O3	0.0467 (17)	0.0581 (18)	0.0335 (14)	0.0199 (13)	0.0092 (13)	−0.0027 (12)
O4	0.062 (2)	0.093 (2)	0.0409 (17)	0.0372 (17)	0.0136 (15)	0.0048 (16)
O5	0.0358 (14)	0.0282 (13)	0.0376 (13)	0.0012 (9)	0.0187 (11)	0.0010 (10)
N1	0.0268 (15)	0.0249 (15)	0.0291 (14)	0.0013 (11)	0.0150 (12)	0.0001 (12)
N2	0.0290 (16)	0.0272 (15)	0.0324 (15)	−0.0013 (11)	0.0167 (13)	−0.0025 (12)
N3	0.0333 (16)	0.0270 (15)	0.0330 (15)	−0.0004 (11)	0.0191 (13)	−0.0013 (12)
N4	0.0294 (16)	0.0237 (14)	0.0335 (15)	0.0017 (11)	0.0174 (13)	−0.0004 (12)
N5	0.0347 (17)	0.0301 (16)	0.0369 (16)	0.0007 (12)	0.0219 (14)	−0.0036 (13)
N6	0.0358 (17)	0.0252 (15)	0.0318 (15)	0.0014 (11)	0.0178 (13)	0.0011 (12)
C1	0.0235 (17)	0.0256 (17)	0.0263 (16)	0.0031 (12)	0.0136 (14)	0.0018 (13)
C2	0.0246 (17)	0.0237 (17)	0.0288 (17)	0.0001 (13)	0.0134 (14)	−0.0015 (14)
C3	0.037 (2)	0.0250 (18)	0.0401 (19)	0.0033 (14)	0.0245 (17)	−0.0003 (15)
C4	0.038 (2)	0.0248 (18)	0.047 (2)	−0.0018 (14)	0.0256 (18)	−0.0050 (16)
C5	0.038 (2)	0.033 (2)	0.046 (2)	−0.0044 (15)	0.0284 (18)	0.0015 (17)
C6	0.033 (2)	0.0290 (19)	0.043 (2)	0.0001 (14)	0.0272 (17)	−0.0014 (16)
C7	0.0256 (18)	0.0288 (18)	0.0273 (17)	−0.0017 (13)	0.0140 (14)	−0.0012 (14)
C8	0.0295 (19)	0.0311 (19)	0.0331 (18)	−0.0030 (14)	0.0190 (16)	−0.0054 (15)
C9	0.063 (3)	0.032 (2)	0.077 (3)	−0.0054 (18)	0.055 (2)	−0.005 (2)
C10	0.059 (3)	0.050 (3)	0.083 (3)	−0.007 (2)	0.055 (3)	−0.003 (2)
C11	0.030 (2)	0.049 (2)	0.043 (2)	−0.0001 (16)	0.0234 (17)	−0.0061 (18)
C12	0.035 (2)	0.037 (2)	0.038 (2)	0.0036 (15)	0.0193 (17)	−0.0041 (16)
C13	0.0281 (18)	0.0252 (17)	0.0252 (16)	0.0002 (13)	0.0129 (14)	0.0029 (13)
C14	0.0269 (18)	0.0264 (18)	0.0262 (17)	0.0032 (13)	0.0098 (14)	0.0015 (14)
C15	0.033 (2)	0.0308 (19)	0.0366 (19)	−0.0023 (14)	0.0190 (16)	0.0015 (16)
C16	0.043 (2)	0.031 (2)	0.042 (2)	−0.0090 (16)	0.0199 (19)	0.0012 (16)
C17	0.049 (2)	0.0262 (19)	0.046 (2)	−0.0033 (16)	0.0270 (19)	−0.0035 (17)
C18	0.045 (2)	0.0263 (19)	0.039 (2)	0.0017 (15)	0.0227 (18)	−0.0018 (15)
C19	0.032 (2)	0.033 (2)	0.0356 (19)	0.0016 (15)	0.0172 (16)	−0.0041 (16)
C20	0.047 (3)	0.050 (2)	0.062 (3)	0.0093 (18)	0.034 (2)	−0.007 (2)
C21	0.036 (2)	0.030 (2)	0.037 (2)	0.0042 (15)	0.0128 (18)	−0.0031 (16)
C22	0.066 (3)	0.085 (4)	0.077 (3)	0.013 (3)	0.034 (3)	0.006 (3)
O6	0.0402 (16)	0.0272 (14)	0.0627 (17)	0.0008 (10)	0.0089 (13)	−0.0022 (12)

### Geometric parameters (Å, °)

**Table:**

Mn1—O3	2.113 (2)	C5—C6	1.385 (4)
Mn1—O5	2.245 (2)	C5—H5	0.9500
Mn1—O1	2.284 (2)	C6—H6	0.9500
Mn1—O2	2.295 (2)	C7—C8	1.487 (4)
Mn1—N1	2.298 (3)	C8—C9	1.375 (5)
Mn1—N4	2.387 (3)	C9—C10	1.380 (5)
Mn1—N6	2.393 (3)	C9—H9	0.9500
Mn1—C19	2.635 (4)	C10—C11	1.370 (5)
O1—C19	1.250 (4)	C10—H10	0.9500
O2—C19	1.269 (4)	C11—C12	1.380 (5)
O3—C21	1.246 (4)	C11—H11	0.9500
O4—C21	1.235 (4)	C12—H12	0.9500
O5—H5A	0.8400	C13—C14	1.494 (4)
O5—H5B	0.8400	C14—C15	1.383 (4)
N1—C13	1.328 (4)	C15—C16	1.384 (4)
N1—C1	1.329 (4)	C15—H15	0.9500
N2—C7	1.334 (4)	C16—C17	1.366 (5)
N2—C1	1.337 (4)	C16—H16	0.9500
N3—C13	1.332 (4)	C17—C18	1.384 (5)
N3—C7	1.349 (4)	C17—H17	0.9500
N4—C6	1.335 (4)	C18—H18	0.9500
N4—C2	1.349 (4)	C19—C20	1.498 (5)
N5—C12	1.330 (4)	C20—H20A	0.9800
N5—C8	1.343 (4)	C20—H20B	0.9800
N6—C18	1.331 (4)	C20—H20C	0.9800
N6—C14	1.344 (4)	C21—C22	1.514 (5)
C1—C2	1.478 (4)	C22—H22A	0.9800
C2—C3	1.381 (4)	C22—H22B	0.9800
C3—C4	1.375 (4)	C22—H22C	0.9800
C3—H3	0.9500	O6—H6A	0.8400
C4—C5	1.377 (4)	O6—H6B	0.8400
C4—H4	0.9500		
			
O3—Mn1—O5	169.93 (10)	N4—C6—C5	123.1 (3)
O3—Mn1—O1	96.91 (11)	N4—C6—H6	118.5
O5—Mn1—O1	93.07 (9)	C5—C6—H6	118.5
O3—Mn1—O2	97.49 (9)	N2—C7—N3	125.2 (3)
O5—Mn1—O2	89.08 (8)	N2—C7—C8	117.0 (3)
O1—Mn1—O2	57.06 (8)	N3—C7—C8	117.7 (3)
O3—Mn1—N1	84.55 (9)	N5—C8—C9	122.7 (3)
O5—Mn1—N1	86.20 (8)	N5—C8—C7	116.2 (3)
O1—Mn1—N1	148.84 (9)	C9—C8—C7	121.2 (3)
O2—Mn1—N1	153.86 (9)	C8—C9—C10	118.5 (3)
O3—Mn1—N4	91.75 (9)	C8—C9—H9	120.7
O5—Mn1—N4	88.54 (8)	C10—C9—H9	120.7
O1—Mn1—N4	80.48 (9)	C11—C10—C9	119.8 (4)
O2—Mn1—N4	137.27 (9)	C11—C10—H10	120.1
N1—Mn1—N4	68.35 (9)	C9—C10—H10	120.1
O3—Mn1—N6	86.51 (10)	C10—C11—C12	117.7 (3)
O5—Mn1—N6	86.38 (9)	C10—C11—H11	121.1
O1—Mn1—N6	142.68 (9)	C12—C11—H11	121.1
O2—Mn1—N6	85.63 (9)	N5—C12—C11	123.9 (3)
N1—Mn1—N6	68.43 (9)	N5—C12—H12	118.1
N4—Mn1—N6	136.71 (9)	C11—C12—H12	118.1
O3—Mn1—C19	96.82 (10)	N1—C13—N3	124.5 (3)
O5—Mn1—C19	92.61 (9)	N1—C13—C14	116.0 (3)
O1—Mn1—C19	28.32 (9)	N3—C13—C14	119.4 (3)
O2—Mn1—C19	28.80 (9)	N6—C14—C15	123.2 (3)
N1—Mn1—C19	176.91 (10)	N6—C14—C13	114.7 (3)
N4—Mn1—C19	108.78 (10)	C15—C14—C13	122.2 (3)
N6—Mn1—C19	114.37 (10)	C14—C15—C16	118.7 (3)
C19—O1—Mn1	91.6 (2)	C14—C15—H15	120.7
C19—O2—Mn1	90.6 (2)	C16—C15—H15	120.7
C21—O3—Mn1	146.0 (2)	C17—C16—C15	118.7 (3)
Mn1—O5—H5A	115.1	C17—C16—H16	120.6
Mn1—O5—H5B	117.1	C15—C16—H16	120.6
H5A—O5—H5B	107.7	C16—C17—C18	119.1 (3)
C13—N1—C1	116.3 (3)	C16—C17—H17	120.5
C13—N1—Mn1	122.0 (2)	C18—C17—H17	120.5
C1—N1—Mn1	121.7 (2)	N6—C18—C17	123.5 (3)
C7—N2—C1	114.7 (3)	N6—C18—H18	118.3
C13—N3—C7	114.7 (3)	C17—C18—H18	118.3
C6—N4—C2	117.3 (3)	O1—C19—O2	120.4 (3)
C6—N4—Mn1	123.9 (2)	O1—C19—C20	120.2 (3)
C2—N4—Mn1	118.8 (2)	O2—C19—C20	119.3 (3)
C12—N5—C8	117.4 (3)	O1—C19—Mn1	60.06 (17)
C18—N6—C14	116.9 (3)	O2—C19—Mn1	60.56 (17)
C18—N6—Mn1	124.3 (2)	C20—C19—Mn1	174.2 (3)
C14—N6—Mn1	118.8 (2)	C19—C20—H20A	109.5
N1—C1—N2	124.5 (3)	C19—C20—H20B	109.5
N1—C1—C2	116.6 (3)	H20A—C20—H20B	109.5
N2—C1—C2	118.9 (3)	C19—C20—H20C	109.5
N4—C2—C3	123.2 (3)	H20A—C20—H20C	109.5
N4—C2—C1	114.5 (3)	H20B—C20—H20C	109.5
C3—C2—C1	122.3 (3)	O4—C21—O3	124.0 (3)
C4—C3—C2	118.4 (3)	O4—C21—C22	118.6 (3)
C4—C3—H3	120.8	O3—C21—C22	117.2 (3)
C2—C3—H3	120.8	C21—C22—H22A	109.5
C3—C4—C5	119.4 (3)	C21—C22—H22B	109.5
C3—C4—H4	120.3	H22A—C22—H22B	109.5
C5—C4—H4	120.3	C21—C22—H22C	109.5
C4—C5—C6	118.7 (3)	H22A—C22—H22C	109.5
C4—C5—H5	120.7	H22B—C22—H22C	109.5
C6—C5—H5	120.7	H6A—O6—H6B	104.7
			
O3—Mn1—O1—C19	91.6 (2)	C6—N4—C2—C1	178.7 (3)
O5—Mn1—O1—C19	−89.8 (2)	Mn1—N4—C2—C1	−0.6 (3)
O2—Mn1—O1—C19	−2.90 (19)	N1—C1—C2—N4	−1.4 (4)
N1—Mn1—O1—C19	−177.55 (19)	N2—C1—C2—N4	−179.7 (3)
N4—Mn1—O1—C19	−177.8 (2)	N1—C1—C2—C3	178.2 (3)
N6—Mn1—O1—C19	−1.7 (3)	N2—C1—C2—C3	−0.2 (5)
O3—Mn1—O2—C19	−90.5 (2)	N4—C2—C3—C4	0.8 (5)
O5—Mn1—O2—C19	97.1 (2)	C1—C2—C3—C4	−178.7 (3)
O1—Mn1—O2—C19	2.86 (18)	C2—C3—C4—C5	0.4 (5)
N1—Mn1—O2—C19	176.6 (2)	C3—C4—C5—C6	−1.6 (5)
N4—Mn1—O2—C19	10.3 (2)	C2—N4—C6—C5	−0.4 (5)
N6—Mn1—O2—C19	−176.4 (2)	Mn1—N4—C6—C5	178.8 (2)
O5—Mn1—O3—C21	145.7 (5)	C4—C5—C6—N4	1.6 (5)
O1—Mn1—O3—C21	−42.1 (5)	C1—N2—C7—N3	1.2 (4)
O2—Mn1—O3—C21	15.4 (5)	C1—N2—C7—C8	−177.5 (3)
N1—Mn1—O3—C21	169.2 (5)	C13—N3—C7—N2	−2.3 (4)
N4—Mn1—O3—C21	−122.8 (5)	C13—N3—C7—C8	176.4 (3)
N6—Mn1—O3—C21	100.5 (5)	C12—N5—C8—C9	−2.0 (5)
C19—Mn1—O3—C21	−13.6 (5)	C12—N5—C8—C7	176.9 (3)
O3—Mn1—N1—C13	−85.8 (2)	N2—C7—C8—N5	5.5 (4)
O5—Mn1—N1—C13	90.2 (2)	N3—C7—C8—N5	−173.4 (3)
O1—Mn1—N1—C13	179.9 (2)	N2—C7—C8—C9	−175.6 (3)
O2—Mn1—N1—C13	10.1 (4)	N3—C7—C8—C9	5.6 (5)
N4—Mn1—N1—C13	−179.9 (3)	N5—C8—C9—C10	1.7 (6)
N6—Mn1—N1—C13	2.6 (2)	C7—C8—C9—C10	−177.2 (3)
O3—Mn1—N1—C1	91.8 (2)	C8—C9—C10—C11	−0.3 (6)
O5—Mn1—N1—C1	−92.2 (2)	C9—C10—C11—C12	−0.7 (6)
O1—Mn1—N1—C1	−2.5 (3)	C8—N5—C12—C11	1.0 (5)
O2—Mn1—N1—C1	−172.3 (2)	C10—C11—C12—N5	0.3 (5)
N4—Mn1—N1—C1	−2.2 (2)	C1—N1—C13—N3	−0.1 (5)
N6—Mn1—N1—C1	−179.8 (2)	Mn1—N1—C13—N3	177.7 (2)
O3—Mn1—N4—C6	98.8 (3)	C1—N1—C13—C14	178.9 (3)
O5—Mn1—N4—C6	−91.3 (3)	Mn1—N1—C13—C14	−3.3 (4)
O1—Mn1—N4—C6	2.1 (2)	C7—N3—C13—N1	1.7 (4)
O2—Mn1—N4—C6	−4.3 (3)	C7—N3—C13—C14	−177.3 (3)
N1—Mn1—N4—C6	−177.8 (3)	C18—N6—C14—C15	0.1 (4)
N6—Mn1—N4—C6	−174.5 (2)	Mn1—N6—C14—C15	179.4 (2)
C19—Mn1—N4—C6	1.0 (3)	C18—N6—C14—C13	−178.9 (3)
O3—Mn1—N4—C2	−82.1 (2)	Mn1—N6—C14—C13	0.4 (3)
O5—Mn1—N4—C2	87.9 (2)	N1—C13—C14—N6	1.8 (4)
O1—Mn1—N4—C2	−178.8 (2)	N3—C13—C14—N6	−179.2 (3)
O2—Mn1—N4—C2	174.93 (19)	N1—C13—C14—C15	−177.2 (3)
N1—Mn1—N4—C2	1.4 (2)	N3—C13—C14—C15	1.8 (4)
N6—Mn1—N4—C2	4.7 (3)	N6—C14—C15—C16	−0.1 (5)
C19—Mn1—N4—C2	−179.9 (2)	C13—C14—C15—C16	178.8 (3)
O3—Mn1—N6—C18	−96.7 (3)	C14—C15—C16—C17	0.3 (5)
O5—Mn1—N6—C18	90.5 (3)	C15—C16—C17—C18	−0.5 (5)
O1—Mn1—N6—C18	0.1 (3)	C14—N6—C18—C17	−0.3 (5)
O2—Mn1—N6—C18	1.1 (3)	Mn1—N6—C18—C17	−179.6 (3)
N1—Mn1—N6—C18	177.8 (3)	C16—C17—C18—N6	0.5 (5)
N4—Mn1—N6—C18	174.5 (2)	Mn1—O1—C19—O2	5.1 (3)
C19—Mn1—N6—C18	−0.8 (3)	Mn1—O1—C19—C20	−173.3 (3)
O3—Mn1—N6—C14	84.0 (2)	Mn1—O2—C19—O1	−5.1 (3)
O5—Mn1—N6—C14	−88.8 (2)	Mn1—O2—C19—C20	173.3 (3)
O1—Mn1—N6—C14	−179.1 (2)	O3—Mn1—C19—O1	−91.9 (2)
O2—Mn1—N6—C14	−178.2 (2)	O5—Mn1—C19—O1	91.6 (2)
N1—Mn1—N6—C14	−1.5 (2)	O2—Mn1—C19—O1	174.9 (3)
N4—Mn1—N6—C14	−4.8 (3)	N4—Mn1—C19—O1	2.3 (2)
C19—Mn1—N6—C14	179.9 (2)	N6—Mn1—C19—O1	178.84 (19)
C13—N1—C1—N2	−1.2 (4)	O3—Mn1—C19—O2	93.1 (2)
Mn1—N1—C1—N2	−178.9 (2)	O5—Mn1—C19—O2	−83.31 (19)
C13—N1—C1—C2	−179.4 (3)	O1—Mn1—C19—O2	−174.9 (3)
Mn1—N1—C1—C2	2.8 (4)	N4—Mn1—C19—O2	−172.65 (18)
C7—N2—C1—N1	0.6 (4)	N6—Mn1—C19—O2	3.9 (2)
C7—N2—C1—C2	178.9 (3)	Mn1—O3—C21—O4	−167.0 (3)
C6—N4—C2—C3	−0.9 (5)	Mn1—O3—C21—C22	18.2 (7)
Mn1—N4—C2—C3	179.9 (2)		

### Hydrogen-bond geometry (Å, °)

**Table:**

*D*—H···*A*	*D*—H	H···*A*	*D*···*A*	*D*—H···*A*
O5—H5A···N5^i^	0.84	2.16	2.924 (3)	151.
O5—H5B···O6^ii^	0.84	1.88	2.704 (3)	168.
O6—H6A···O2	0.84	1.95	2.791 (3)	175.
O6—H6B···O4^iii^	0.84	1.87	2.711 (4)	176.
C3—H3···O5^iv^	0.95	2.46	3.399 (4)	170.
C5—H5···O3^i^	0.95	2.59	3.338 (4)	136.
C6—H6···O1	0.95	2.33	2.983 (4)	125.
C18—H18···O2	0.95	2.55	3.198 (4)	125.

Symmetry codes: (i) *x*, −*y*+1/2, *z*−1/2; (ii) −*x*+1, −*y*, −*z*; (iii) −*x*+2, −*y*, −*z*+1; (iv) *x*, −*y*+1/2, *z*+1/2.
